# Clinical pharmacology strategies in supporting drug development and approval of antibody–drug conjugates in oncology

**DOI:** 10.1007/s00280-021-04250-0

**Published:** 2021-04-01

**Authors:** Stephanie N. Liu, Chunze Li

**Affiliations:** Department of Clinical Pharmacology, Genentech, Inc, 1 DNA Way, South San Francisco, CA 94080 USA

**Keywords:** Antibody–drug conjugate, Clinical pharmacology, Population pharmacokinetics, Exposure–response analysis, Specific population, Drug interaction, QTc prolongation

## Abstract

Antibody–drug conjugates (ADCs) are important molecular entities in the treatment of cancer. These conjugates combine the target specificity of monoclonal antibodies with the potent anti-cancer activity of small-molecule therapeutics. The complex structure of ADCs poses unique challenges to characterize the drug’s pharmacokinetics (PKs) and pharmacodynamics (PDs) since it requires a quantitative understanding of the PK and PD properties of multiple different molecular species (e.g., ADC conjugate, total antibody and unconjugated cytotoxic drug). As a result, clinical pharmacology strategy of an ADC is rather unique and dependent on the linker/cytotoxic drug technology, heterogeneity of the ADC, PK and safety/efficacy profile of the specific ADC in clinical development. In this review, we summarize the clinical pharmacology strategies in supporting development and approval of ADCs using the approved ADCs as specific examples to illustrate the customized approach to clinical pharmacology assessments in their clinical development.

## Introduction

Antibody drug conjugates (ADCs) are an emerging class of anti-cancer therapeutic agents that combine the antigen targeting specificity and favorable pharmacokinetic properties of monoclonal antibodies (mAbs) with the cytotoxic potential of small-molecule chemotherapeutics [1]. ADCs typically consist of three components, namely a mAb to determine which cells to be targeted, a cytotoxic drug to determine the mechanism of action by which cells are killed, and a chemical linker that attaches these two components together to determine how the drug is released. The mAb component of an ADC enables the ADC to specifically bind to targeted cell surface antigens overexpressed on the tumor cells. Upon binding, the ADCs are internalized and trafficked to lysosomes, from which the cytotoxic drug is released within the cell, thus resulting in the cell death. The use of targeted delivery of highly potent cytotoxic drugs is designed to enhance the antitumor effects of the molecule while minimizing the toxicity in the normal tissues.

As of January 2020, nine ADCs have received US Food and Drug Administration (FDA) approval [2]. The first of these, (1) gemtuzumab ozogamicin (Mylotarg®; an anti-CD33 mAb linked to calicheamicin), for the treatment of acute myelogenous leukemia (AML) was approved in 2000 under the FDA accelerated-approval process [3]. In 2010, this agent was voluntarily withdrawn from the market due to confirmatory trials failing to demonstrate clinical benefit and safety concerns [3]. Gemtuzumab ozogamicin was re-approved in 2018 at a sub-fractionated dose of 3–6 mg/m^2^ (compared to 9 mg/m^2^ at first approval) [4]. Since gemtuzumab ozogamicin’s initial market approval, seven more ADCs were FDA approved: (2) brentuximab vedotin (Adcetris®; an anti-CD30 mAb and monomethyl auristatin E [MMAE] conjugate) for the treatment of Hodgkin lymphoma and systemic anaplastic large-cell lymphoma, (3) trastuzumab emtansine (T-DM1, Kadcyla®; an anti-human epidermal growth factor receptor 2 (HER2) mAb and DM1 [a derivative of maytansine] conjugate) for the treatment of HER2 + metastatic breast cancer (mBC), (4) inotuzumab ozogamicin (Besponsa®, an anti-CD22 mAb and calicheamicin conjugate) for the treatment of adults with relapsed or refractory B-cell precursor acute lymphoblastic leukemia (ALL), (5) polatuzumab vedotin (Polivy®, an anti-CD79b mAb and MMAE conjugate) for the treatment of relapsed or refractory diffuse large B-cell lymphoma (DLBCL), (6) enfortumab vedotin (Padcev®, an anti-Nectin 4 mAb and MMAE conjugate) for the treatment of locally advanced or metastatic urothelial cancer, (7) trastuzumab deruxtecan (Enhertu®, an anti-HER2 mAb and exatecan derivative conjugate) for the treatment of HER2 + mBC, and (8) sacituzumab govitegcan (Trodelvy®, an anti-Trop-2 mAb and SN-38 conjugate) for the treatment of metastatic triple-negative breast cancer [5–11]. In August 2020, the 9th ADC, namely belantamab mafodotin-blmf (Blenrep®, an anti-BCMA mAb and MMAF conjugate) achieved accelerated approval from FDA for the treatment relapsed and refractory multiple myeloma [12].

These ADCs prove that the therapeutic window of otherwise intolerable cytotoxic drugs can be improved to a therapeutically beneficial level by conjugating it to an antibody. Despite the great success of ADCs, it is worth noting that the therapeutic window for ADCs remains relatively narrow with the maximum tolerated dose (MTD) often reached before ADCs achieve the maximal efficacious dose [13]. As a result, numerous innovative approaches (e.g., site-specific conjugation or novel payloads) have been implemented to further improve the therapeutic window, resulting in the “next-generation” ADCs, many of which are currently tested in clinical development.

The current understanding of the mechanism at which ADCs are cleared is through two major pathways: proteolytic degradation and deconjugation [14, 15]. ADC clearance through proteolytic degradation is driven primarily by catabolism mediated by target-specific or nonspecific cellular uptake followed by lysosomal degradation, similar to mAbs. Deconjugation clearance is usually mediated by enzymatic or chemical cleavage (e.g., maleimide exchange) of the linker leading to the release of the cytotoxic drug from the ADC [16]. Once released from the ADC, the cytotoxic drug may be further metabolized, transported, and eliminated via traditional mechanisms applicable to small molecules (see DDI section). Alternatively, ADC catabolism and deconjugation in vivo leads to the formation of multiple different molecular species (e.g., ADC species with different drug antibody ratios [DAR]) and payload-containing catabolites) [17]. The bioanalytical strategy for ADCs thus requires defining the specific analytes of relevance to clinical pharmacology. Although multiple analytes may be quantified following the dosing of an ADC, the clinical importance of the multi-analyte bioanalytical data in context of safety and efficacy remains to be established. With numerous ADCs in clinical development, streamlining the bioanalytical and clinical pharmacology strategy is critical.

**Fig. 1:**
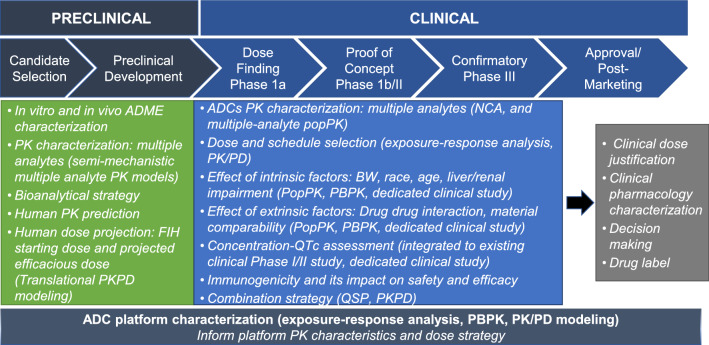
Clinical pharmacology considerations in the stages of ADC drug development

The clinical pharmacology assessments for ADCs to address scientific and regulatory concerns are summarized in Fig. 1. The clinical pharmacology of an ADC incorporates elements of small molecule and mAb (large molecule) development strategies. The scope of work is usually unique and dependent on the linker/cytotoxic drug technology, heterogeneity of the ADC, pharmacokinetics (PK) and safety/efficacy profile of the specific ADC in clinical development. Given the structural complexity, multiple analytes were measured across Phase I, Phase II and Phase III clinical studies to characterize the PK of an ADC, including, but not limited to, conjugate, total antibody and unconjugated payload. Intrinsic (e.g., body weight, organ dysfunction) and extrinsic factors (e.g., concomitant medications) likely to impact the PK of an ADC were assessed using diverse approaches (Fig. 1). Relationships between exposure to ADC conjugate (and other relevant analytes) and efficacy/safety were assessed in support of the clinical dosing regimen selection using quantitative approaches. This review summarized the unique clinical pharmacology consideration in supporting development and approval of ADCs (Fig. 1). The seven approved ADCs are used as specific examples to illustrate the customized approach to clinical pharmacology assessments in their clinical development. Sacituzumab govitecan and belantamab mafodotin-blmf were not included in the summary due to limited clinical pharmacology information available at the time of the review.

## Bioanalytical consideration

ADCs incorporate both large- and small-molecule characteristics and are usually present as a heterogeneous mixture of the species differing not only in the number of cytotoxic drugs attached to the antibody, but also in the protein conjugation sites of drug linkage [18]. Furthermore, biotransformations in vivo can lead to additional changes in DARs resulting in dynamically changing mixtures. As a result, unlike mAbs, the heterogeneity of ADCs in vivo makes it critical to measure multiple analytes in clinical trials [17, 19]. These analytes may include, but are not limited to, the following: ADC conjugate (measured as conjugated antibody or conjugated payload), total antibody (TAb, conjugated and unconjugated antibody), unconjugated antibody and unconjugated (free) payload. Conjugated antibody and conjugated payload are the two alternative ways to quantify the ADC conjugate [20]. From the perspective of the antibody, the ADC conjugate can be measured as “conjugated antibody”, namely the concentration of antibody molecules with one or more cytotoxic drugs attached. This bioanalytical method is used to measure serum concentrations of ADC conjugate for brentuximab vedotin, inotuzumab ozogamicin, T-DM1, enfortumab vedotin, and trastuzumab deruxtecan [21–25]. Alternatively, from the perspective of the payload, the ADC conjugate can be measured as “conjugated drug”, namely as the total concentration of cytotoxic drug that is conjugated to the antibody. Currently, only ADCs with cleavable linker are amenable to the conjugated drug assay. This bioanalytical method is used to measure polatuzumab vedotin given that not all the DAR species can be measured accurately in the conjugated-antibody ELISA assay [26]. In comparison, gemtuzumab ozogamicin does not measure ADC conjugate. Instead, gemtuzumab ozogamicin measured TAb and unconjugated calicheamicin [27, 28] likely due to the availability of bioanalytical techniques at the time of development.

Although multiple analytes may be quantified following the dosing of an ADC, the bioanalytical strategy for ADCs requires defining the specific analytes of relevance to clinical pharmacology in the context of safety and efficacy. Most commonly, three analytes, namely ADC conjugate, TAb and unconjugated cytotoxic drug are measured in preclinical and clinical studies to characterize the PK properties of an ADC [17, 19].

## Population PK modeling

Population PK modeling is an important approach to characterize the ADC PK properties and assess the effect of intrinsic and extrinsic factors on ADC PK, and thus guide dose recommendations in specific populations (e.g., geriatric patients or patients with organ dysfunction). Given multiple analytes were measured for an ADC during its clinical development, one of the unique features of population PK for an ADC is that more than one analyte is often included in the population PK model development. ADC conjugate, the main analyte of interest per mechanism of action of ADCs, is the most common analyte included in the population PK model. Additionally, given the high potency of cytotoxic drugs, the potential contribution of unconjugated drug to safety could not be ruled out. Exposure-safety analysis with unconjugated cytotoxic drug has been conducted for the four out of the seven approved ADCs (see exposure–response section). As a result, unconjugated drug analyte is often included in the population PK model in addition to ADC conjugate to understand the PK characteristics of unconjugated drugs after ADC dosing and generate exposure metrics for exposure-response analysis.

As shown in Table 1, five out of the seven approved ADCs include the two analytes in their population PK models. Integrated two-analyte models (i.e., ADC conjugate-unconjugated payload models) were developed for brentuximab vedotin, polatuzumab vedotin, enfortumab vedotin and trastuzumab deruxtecan, while for gemtuzumab ozogamicin population PK model for TAb and unconjugated payload was developed separately and ADC conjugate analyte was not measured clinically [28]. Typically, the ADC conjugate is dosed in the linear range based on the findings of the phase 1 dose escalation study. The population PK model structures for ADC conjugate are usually characterized by a 2- or 3- compartment model with a mixture of linear and non-linear elimination pathways. Notably, three out of the seven ADCs have non-linear time-dependent clearance and all of them target hematological malignancy (Table 1). The ADC linear clearance (CL = 1.6–2.5 L/day) and central volume of distribution (Vc = 6.4–6.7 L) are similar for brentuximab vedotin and enfortumab vedotin, the MMAE-containing ADCs that share the same cytotoxic drug and linker but against different targets [21, 29]. Polatuzumab vedotin is not included in the comparison due to apparent non-linear and time-dependent PK [30]. Conversely, T-DM1 and trastuzumab deruxtecan, both of which share the same mAb but with different cytotoxic drugs and linkers, exhibited linear PK with similar CL (0.4–0.7 L/day) and central volume of distribution (~ 3 L) at the clinical approved dose [23, 31]. As expected for small molecules, the unconjugated payloads released from ADCs exhibit faster apparent clearances (> 19 L/day) from circulation with larger apparent central volume of distribution (> 80 L) into extravascular tissues compared to the ADC or TAb. For MMAE-containing ADCs, the MMAE apparent clearance and apparent central volume of distribution is 45–66 L/day and 80–99 L, respectively. The calicheamicin analyte was not characterized in the population PK model for inotuzumab ozogamicin so PK parameters for the payload were not available for comparison, but for gemtuzumab ozogamicin, unconjugated calicheamicin CL/F was 32 L/day and V1/F was 97 L [32]. For trastuzumab deruxtecan, the unconjugated DXd clearance (CL = 19 L/day) was the lowest for the payloads and the central volume was not estimable with data collected so it was fixed to nonclinical data in the population PK model [25].

**Table 1:** Summary of population PK models of the seven approved ADCs

ADC	Overall model structure	Analyte	Model description	Key populations parameters				Clinical approved dose regimen
				CL(L/day)	V(L)	Covariates^	Others	
Gemtuzumab ozogamicin [4, 32]	Two-analyte (TAb-unconjugated calicheamicin) model	TAb	2-COMP, LE + TD	CL* = 2.81 [2.52–3.096] CL2* = 66 [39.1–92.9] Q = 2.07 [1.51–2.64]	V1 = 6.37 [5.86–6.68] V2 = 8.58 [6.9–10.2]	CL: BW (0.75,FIX), DOSE, ALB V1: BW (0.75, FIX), DOSE, SEX, ALB K_des_: BMAR, BBC, COMB	K_des_ = 18[11.2–25.0]	3 mg/m [2] (with a maximum of one 4.5 mg vial) on Days 1, 4, and 7
		Calicheamicin	2-COMP, LE + 1^st^ Formation	CL/F = 32.4 [25.7–39.1] Q/F = 68.9 [54.2–83.5]	V1/F = 96.9[90.1–104] V2/F = 61.0 [394–824]	CL/F: BW (0.75, FIX) V1/F: BW (1, FIX)	K_f_/F = 0.0262 [0.001–0.0510]	
Brentuximab vedotin [29]	Integrated two-analyte (ADC-unconjugated MMAE) model (joint model)	ADC	3-COMP, LE	CL = 1.56 (2.9%) Q2 = 2.83 (5.95) Q3 = 0.708 (8.2%)	V1 = 4.29 (1.9%) V2 = 3.83 (6.3%) V3 = 9.52 (8.8%)	CL: Q2, Q3, BW (0.698)* V1: BW (0.503), SEX V2, V3: BW (0.503)		1.8 mg/kg q3w, with a maximum of 180 mg for individuals with BW > 100 kg
		Unconjugated MMAE	2-COMP, LE + cycle dependent semi mechanistic formation	CL = 55.7 (5.2%) Q = 65 (30%)	V1 = 79.8 (11%) V2 = 28.1 (14%)	CL, Q: BW(0.75, FIX) V1, V2: BW (1, FIX)	fm by cycle = − 0.261 (14%); beta = 0.0785 (12%)	
Inotuzumab ozogamicin [47]	One analyte model with ADC conjugate	ADC	2-COMP, LE + TD	CL* = 2.71 [2.54–2.88] CL2* = 8.86 [7.97–9.74] Q = 0.972 [0.838–1.11]	V1 = 6.7 [6.40–7.00] V2 = 5.10 [4.60–5.60]	CL: BSA (1.54), ALL, RTX CL2: BSA (1.64) V1: BSA; K_des_: ALL, BLSTPB	K_des_* = 0.809 [0.602–1.02] day 1	0.8 mg/m [2] on day1 followed by 0.5 mg/m [2] on days 8 and 15 Q3W dosing regimen
Trastuzumab emtansine (T-DM1) [31]	One analyte model with ADC conjugate	ADC	2-COMP, LE	CL = 0.676 [0.661–0.691] Q = 1.534 [1.286–1.83]	V1 = 3.127 [3.08–3.174] V2 = 0.66 [0.58–0.752]	CL: BW (0.49), ECD, ALB, TMBD, TBL, AST V1: BW		3.6 mg/kg Q3W
Polatuzumab vedotin [30]	Integrated two-analyte model (ADC-unconjugated MMAE model)	acMMAE	2-COMP, TD LE + TD ELE + MM NLE	CLINF** = 0.83 [0.77–0.88] CLT** = 0.15 [0.092–0.128] Q = 0.348 [0.33–0.367]	V1 = 3.15 [3.05–3.25] V2 = 3.98 [1.75–4.2]	CLINF: BW (0.73, FIX), SEX, ALB, RTX, OB, B-Cells, TMBD CLT: NAÏVE, TMBD, Threshold, B-Cells V1: SEX, ASIAN, NAÏVE Q, V1, V2: BW (0.50, FIX); K_des_: NAÏVE, CT	K_des_** = 0.11 day1; CLINF. MAX = 0.223 [0.185–0.261]; V_max_ = 0.487 [0.035–0.62] ng/mL [31]; Km = 0.604 [0.175–1.03] ng/mL; T50 = 3.53 {3.07–4] months; GAM = 2.27 [1.71–2.82]	1.8 mg/kg Q3W × 6 cycles
		Unconjugated MMAE	2-COMP, L + NL formation, LE + MM NLE	CL = 45.4 [38.2–52.8] Q = 871 [660–1082]	V1 = 82.2 [69.1–95.4] V2 = 200 [176–224]		Refer to Lu et al. [30] for additional information	
Enfortumab vedotin [21]	Integrated two-analyte (ADC-unconjugated MMAE) model	ADC	3-COMP, LE	CL = 2.50 [2.42–2.57] Q2 = 0.0595 [0.0504–0.0701] Q3 = 0.895 [0.845–0.958]	V1 = 3.75 [3.62–3.87] V2 = 4.54 [2.98–5.89] V3 = 2.88 [2.70–3.08]	CL: BW (0.65), AGE, SEX, SOD; Q2, Q3: BW (0.65); V1: BW (0.59), SEX, SOD; V2: BW (0.59); V3: BW (0.59), CT		1.25 mg/kg on days 1, 8, 15 per 28 day cycle, with a maximum of 125 mg for individuals with BW > 100 kg
		Unconjugated MMAE	2-COMP, LE	CL = 65.8 [61.2–69.1] Q2 = 365 [322–418]	V1 = 99.3 [94.0–105] V2 = 118 [107–130]	CL: BW (0.75, FIX), ALB, ECOG, Hgb, Bili; Q2: BW (0.75, FIX); V1: BW (1, FIX), ALB; V2: BW (1, FIX), ALB, Hgb, Race, SEX	DAR = 3.8, FIX; K_des_ = 0.00053 {0.000444–0.000737]	
Trastuzumab deruxtecan [25]	Integrated two-analyte (ADC-unconjugated DXd) model	ADC	2-COMP, LE	CL = 0.42 [0.405–0.434] Q2 = 0.200 [0.190–0.212]	V1 = 2.78 [2.74–2.78] V2 = 5.17 [4.40–5.97]	CL: BW, ALB, TS, SEX, JAP; V1: BW, SEX; V2: JAP		5.4 mg/kg Q3W
		DXd	1-COMP, LE + 1^st^ formation	CL = 19.1 [17.8–20.4]	V = 17^a^BSA (FIX)	CL: BW, RITO, ITRA, AST, BILI, AGE; V: FL-DP2, FL-DP1	K_rel_ = 0.0159 [0.0146–0.172]; K_frac_ > c1 = 0.829 [0.803–0.01721]	

Typical value [95% CI] or typical value (%RSE); ^covariates provided for CL and V parameters only

*ADC* antibody–drug conjugate (measured as conjugated antibody), *acMMAE* conjugated MMAE (measured as conjugated payload), *TAb* total antibody, *T-DM1* trastuzumab emtansine, *MMAE* monomethyl auristatin E, *DXd* deruxtecan, *2-comp* 2-compartment, *3-comp* 3-compartment, *LE* linear elimination, *NLE* non-linear elimination, *TD* time-dependent clearance, *ELE* exponential linear elimination, *MM* Michaelis–Menten, *CL* linear clearance from central compartment, *CLT* initial time-dependent clearance, *CLNS* nonspecific linear clearance after repeated dosing, *CLINF* CLNS at time of infinity, *Q or Q2 or Q3* intercompartmental clearance, *CL/F* apparent clearance, *Q/F* apparent intercompartmental clearance, *V1* volume of distribution in central compartment, *V2 or V3* volume of distribution in peripheral compartment, *ALB* basesline albumin, *BW* body weight, *BSA* body surface area, *BBC* baseline peripheral blast count, *BMAR* baseline percentage blast in bone marrow, *COMB* combination therapy, *ECD* baseline serum human epidermal growth factor receptor 2 shed extracellular domain concentration, *ALL* acute lymphocytic leukemia, *BLSTPB* baseline percentage of blasts in peripheral blood, *TBL* baseline trastuzumab concentration, *TMBD* baseline sum of longest dimension of target lesions, *AST* aspartate aminotransferase, *SOD* sum of tumor diameter, *RTX* rituximab, *OB* obinutuzumab, *B-Cells* B-cell count, *NAÏVE* treatment naïve, *CT* cancer type, *ECOG* Eastern Cooperative Oncology Group performance score, *Hgb* hemoglobin, *Bili* bilirubin, *TS* tumor size, *JAP* country (japanese vs non-japanese), *RITO* ritonavir, *ITRA* itraconzaole, *FL-DP1* frozen liquid drug product 1, *FL-DP2* frozen liquid drug product 2, *K*_*dec*_ the deconjugation rate for acMMAE, *K*_*des*_ decay coefficient associated with time-dependent CL, *K*_*f*_*/F* apparent fraction of unconjugated calicheamicin that appears in circulation following release from gemtuzumab ozogamicin, *K*_*rels*_ the deconjugation rate for DXd, *K*_*frac*_* > c1* fraction of K_rels_ after cycle 1, *Q3W* every 3 weeks, ^a^ volume of the released drug was not estimable from current data so it was fixed to 17 L/m2 (literature documented volume of distribution of exatecan mesylate DX-8951f) and multiplied by individual body surface area

^*^CL = CL1 + CL2 * exp(-kdes*time). **CL = CLT*EXP(-KDES*T) + CLINF*(1 + CLINFEMAX*T50GAM/(T50GAM + TGAM)) + Vmax*V1/(Km + C)

The covariate effects from body weight (BW) or body surface area (BSA) is consistently identified as one of significant covariates on key PK parameters (i.e., CL and/or Vc) in the final popPK models for all the approved ADCs. The exponential of BW effect on CL ranged from 0.49 to 0.75, thus supporting the BW- and BSA- based dosing strategy for ADCs. It was worth noting that BW and BSA were highly correlated, of these two covariates, BW is usually preferred to be included in the model as it is the simpler measure to obtain. Six out of the seven approved ADCs identified BW as a significant covariate in their population PK model except inotuzumab ozogamicin which included BSA (Table 1). Among the seven approved ADCs, five of them utilized BW-based dosing regimen with the two calicheamicin-containing ADCs using BSA-based dosing. Since these agents have relatively narrow therapeutic windows, some of the ADCs (i.e., brentuximab vedotin, enfortumab vedotin) adopted dose capping strategy to further reduce inter-individual variability for the ADC exposure and thus potentially improve the ADC’s safety profiles, particularly for patients with higher BW (i.e., BW > 100 kg) that would achieve higher drug exposure from a weight-based dosing regimen [21, 22]. Notably, no significant PK differences based on age was observed. Some differences in PK parameters with gender was observed, but post-hoc analyses showed it did not have any clinical meaningful effect on ADC exposures and thus did not warrant dosing adjustment based on gender. The impact of extrinsic and intrinsic factors on ADC PK has been discussed previously [33, 34]. Consistent with other biotherapeutics, baseline albumin and disease factors (e.g., tumor burden) were often identified as significant covariates for ADC clearance, however, the magnitude of the effect of these significant covariates on ADC exposure is minimal compared with overall PK variability and therefore the BW- and BSA- based dose without further adjustment for other factors is considered appropriate for ADCs.

## Organ dysfunction studies

The general concept that hepatic impairment may not affect therapeutic proteins PK, including mAbs and ADCs, is being challenged with emerging evidence [34]. Recent publication by Sun et al. showed that of 20 mAbs and 4 ADCs with hepatic impairment data, a decrease in exposure of 1 mAb and 2 ADCs were observed in patients with hepatic impairment. Although the mechanism is unknown, Sun et al. proposes worsening of disease associated with hepatic impairment may increase the elimination of therapeutic proteins through increased competition of FcRn binding with other soluble proteins (i.e. albumin) and target mediated drug disposition. In addition, the liver and kidneys play an important role in elimination of the small-molecule component of an ADC, namely cytotoxic drug once it gets released from the ADC. As a result, impairment of the functions of these organs may result in alteration of ADC and/or cytotoxic drug clearance, leading to exposure changes, which may in turn impact the safety and efficacy. This is especially important given ADCs generally have a relatively narrow therapeutic index. Therefore, assessment of the impact of organ dysfunction on the disposition of ADCs to inform appropriate dosing in these patients is an important component of clinical pharmacology strategy for these molecules.

Table 2 summarizes the impact of liver and kidney function on ADCs PK and their corresponding dosing recommendation for the seven approved ADCs. It is noted that two alternative approaches were used to characterize the impact of organ dysfunction on ADC PK across the seven approved ADCs (1) a dedicated organ dysfunction clinical study or (2) model-based approach using patients with organ dysfunction across clinical studies to determine the effects of clinical PK. As shown in Table 2, three out of seven ADCs conducted dedicated hepatic and/or renal impairment clinical studies: brentuximab vedotin, T-DM1, and enfortumab vedotin. While for the remainder of ADCs, modeling and simulation through popPK has been utilized to assess the organ dysfunction subpopulation across clinical studies. The current ADC model-based approach requires that existing clinical studies allow enrollment of patients with organ impairment.

**Table 2:** Effect of hepatic and renal impairment on ADC PK and dose recommendation for the seven approved ADCs

ADC	Hepatic impairment					Renal impairment				
	Approach	Result	Label recommendation			Approach	Result	Label recommendation		
			Mild	Moderate	Severe			Mild	Moderate	Severe
Gemtuzumab Ozogamicin [4, 32]	PopPK: NCI criteria	No PK effect in mild (*n* = 58) or moderate (*n* = 6) hepatic impairment	Approved dose	Not studied		PopPK: CrCL	No PK effect in mild (*n* = 149) or moderate (*n* = 47) renal impairment	Approved dose		Avoid
Brentuximab vedotin [5, 36]	Clinical study: Child–Pugh	ADC: AUC GMR 0.67 (90% CI 0.48–0.93) for any hepatic impairment (*n* = 7)	1.8—> 1.2 mg/kg	Avoid		Clinical Study: CrCL	ADC: No PK effect in mild (*n* = 4) or moderate (*n* = 3) renal impairment; AUC GMR 0.71 (90% CI 0.54–0.94) in severe renal impairment (*n* = 3)	Approved dose		Avoid
		MMAE: AUC GMR 2.29 (90% CI 1.27–4.12) for any hepatic impairment (*n* = 7)					MMAE: No PK effect in mild (*n* = 4) or moderate (*n* = 3) renal impairment; AUC GMR 1.90 (90% CI 0.85–4.21) in severe renal impairment (*n* = 3)			
Trastuzumab emtansine (T-DM1) [8, 37]	Clinical study: Child–Pugh	T-DM1: AUC at cycle 1 was 38% and 67% lower in the mild (*n* = 10) and moderate (*n* = 8) hepatic impairment and AUC at cycle 3 was within range of normal hepatic function	Approved dose		Not studied	PopPK: CrCL	No effect on PK in mild (*n* = 254) or moderate (*n* = 53) renal impairment	Approved dose		Not studied
		DM1 and DM1-containing catabolites: low and comparable with or without hepatic impairment								
Inotuzumab ozogamicin [6, 24]	PopPK: NCI criteria	ADC only: CL not impacted by mild (*n* = 150), moderate (*n* = 3), or severe (*n* = 1) hepatic impairment	Approved dose	Not studied		PopPK: CrCL	ADC only: No effect on PK in mild (*n* = 237), moderate (*n* = 122), or severe (*n* = 4) renal impairment	Approved dose		
Polatuzumab vedotin [10, 28]	PopPK: NCI criteria	acMMAE: comparable exposure	Approved dose	Avoid		PopPK: CrCL	No effect on PK in mild (*n* = 161) or moderate (*n* = 109) renal impairment	Approved dose		Not studied
		MMAE: AUC was 40% higher and 37% for Cmax in mild hepatic impairment (*n* = 54); insufficient information in moderate (*n* = 2) and severe (*n* = 0) hepatic impairment								
Enfortumab vedotin [9, 21]	PopPK: NCI criteria	No effect on PK in mild (*n* = 31) hepatic impairment	Approved dose	Avoid		Clinical Study: CrCL and PopPK:CrCL	No effect on PK in mild (*n* = 135), moderate (*n* = 147), or severe (*n* = 8) renal impairment	Approved dose		
Trastuzumab deruxtecan [7, 25]	PopPK: NCI criteria	No effect on PK in mild (*n* = 215) hepatic impairment	Approved dose		Not studied	PopPK: CrCL	No effect on PK in mild (*n* = 206) or moderate (*n* = 58) renal impairment	Approved dose		Not studied

*ADC* antibody–drug conjugate (measured as conjugated antibody), *acMMAE* conjugated MMAE (measured as conjugated payload), *T-DM1* trastuzumab emtansine, *DM1* emtansine, *MMAE* monomethyl auristatin E, *acMMAE* conjugated MMAE, *PK* pharmacokinetics, *CrCL* creatinine clearance, *PopPK* population pharmacokinetics, *NCI* National Cancer Institute

ADC conjugate exposure was generally comparable between patients with hepatic impairment and normal hepatic function for most of the approved ADCs, except for brentuximab vedotin and T-DM1 (Table 2). For brentuximab vedotin, ADC conjugate exposure (i.e., AUC) decreased by 35% in lymphoma patients with moderate hepatic impairment, and there was only one patient each with mild or severe hepatic impairment [35]. For T-DM1, AUC of T-DM1 conjugate at Cycle 1 in patients with mild and moderate hepatic impairment were approximately 38% and 67% lower than that of patients with normal hepatic function, respectively [36]. Interestingly, the exposure difference was less apparent after repeated dosing with T-DM1 AUC at Cycle 3 in patients with mild and moderate hepatic impairment largely comparable to the patients with normal hepatic function. There was no apparent effect of hepatic impairment on the cytotoxic drug exposure except for brentuximab vedotin (unconjugated MMAE AUC GMR 2.29 for any hepatic impairment vs normal hepatic function) and polatuzumab vedotin (unconjugated MMAE AUC GMR 1.40 for mild hepatic impairment vs normal hepatic function) [26, 36]. The fact that the exposure of unconjugated MMAE was increased by two-to-threefold in moderate hepatic impaired patients resulted in label recommendation for brentuximab vedotin to avoid use in patients with moderate to severe hepatic impairment [5]. The comparable unconjugated DM1 exposure and transient change of T-DM1 conjugate exposure in mild or moderate hepatic impairment lead to label recommendation of no adjustments of the dose of T-DM1 in these patients [8, 37]. Although an increase in unconjugated MMAE exposure for polatuzumab vedotin was observed, based on the exposure-safety relationship established across clinical studies, the increased unconjugated MMAE exposure was not clinically relevant and no adjustment in the starting dose is required for polatuzumab vedotin in patients with mild hepatic impairment [26].

For patients with renal impairment, ADC conjugate and cytotoxic drug PK are comparable for most of the approved ADCs, except for brentuximab vedotin in patients with severe renal impairment (ADC AUC GMR 0.71 and MMAE AUC GMR 1.90) (Table 2) [36]. The altered PK in brentuximab vedotin results in label recommendation to avoid use in patients with severe renal impairment [5].

The approach to evaluate organ dysfunction for ADC drug development remains situation dependent, but is trending toward a modeling and simulation approach. The population PK approach is routinely conducted to evaluate the impact of organ dysfunction on the exposure of ADC and its relevant analytes. If there is an impact on the exposure, such an impact on dose recommendation should be assessed in the context of benefit risk assessment and/or exposure–response relationship. In the future, physiologically based pharmacokinetic (PBPK) modeling approach may be used to assess the impact of organ dysfunction on ADC PK once the ADC PBPK model and organ dysfunction patient population is fully established.

## Drug–drug interactions

Assessing drug–drug interaction (DDI) risk associated with ADCs needs to consider both the large- and small-molecule components of the ADC. The cytotoxic payloads, upon release from ADCs, are expected to behave like small molecules and thus may be of concern for enzyme or transporter-mediated DDIs. The FDA and European Medicines Agency (EMA) have issued comprehensive recommendations for in vitro and in vivo studies to evaluate DDI potential for small molecules, but specific guidelines on DDI risk assessment for ADCs have not been issued. Given the relatively high potency and low systemic exposure of cytotoxic payloads, some unique DDI consideration might be needed for ADCs. Different from other molecules, human mass balance study is usually not conducted for most of the approved ADCs (6 out of 7 approved ADCs). Brentuximab vedotin is the only ADC that conducted a clinical excretion study but without complete recovery [21]. Instead, leveraging preclinical ADME data is the main strategy for initial DDI assessment of ADCs.

DDIs related to the payload have been extensively evaluated during the clinical development of an ADC. Table 3 summarizes the approaches, key findings and its implication on the drug label of payload-mediated DDIs for the seven approved ADCs, which include four different payloads: calicheamicin, MMAE, DM1, and DXd. Multiple approaches, namely dedicated clinical DDI study, theoretical risk assessment, physiologically based pharmacokinetic (PBPK) model, concomitant medication analysis, and referencing existing DDI data from a previously established ADC were used for DDI risk assessment. Theoretical risk assessment based on the in vitro DDI and clinical data is the most commonly used approach for the 7 ADCs (Table 3). Dedicated clinical DDI studies were conducted for two out of the seven ADCs: brentuximab vedotin and trastuzumab deruxtecan. PBPK modeling approach by leveraging available clinical DDI data for the same payload was used to inform DDI risk for polatuzumab vedotin, while exploratory concomitant medications analysis using NCA or population PK of clinical data to evaluate the effect of concomitant medications on payload PK was used for T-DM1.

**Table 3:** Payload-mediated DDI and its impact on drug label for the seven approved ADCs

Molecule	Payload	Approach	Victim		Perpetrator		Label Recommendation
			Transporter	Enzyme	Transporter	Enzyme	
Gemtuzumab ozogamicin [4, 28]	Calicheamicin	In vitro, in vivo (rats)	NA	Non-enzymatic reduction; expect no effect from CYP or UGT perpetrators (inhibitors or inducers) on drug CL	Inhibits OATP1B1- OATP1B3; no effect on P-gp, BCRP, OAT1, OAT3, or OCT2	Inhibits UGT1A1; no effect on CYP3A4; no inhibition on CYP1A2, CYP2B6, UGT1A4, UGT1A6, UGT1A9, UGT2B7	No recommendation
Brentuximab vedotin [5, 38]	MMAE	In vitro	P-gp substrate	Substrate of CYP3A4/5	NA	Inhibits CYP3A4/5 but no other CYP isoforms. Did not induce any major CYP450 enzymes in primary cultures of human hepatocytes	Concomitant use of strong CYP3A4 inhibitors or inducers, or P-gp inhibitors, has the potential to affect the exposure to MMAE
		Clinical study	NA	Ketoconazole (CYP3A4/P-gp inhibitor) increased MMAE AUC_0-INF_ by 34% Rifampin (CYP3A4/P-gp inducer) decreased MMAE AUC_0-INF_ by 46%	NA	No effect on midazolam (CYP3A4 substrate) exposure	
Trastuzumab emtansine (T-DM1) [8, 40]	DM1	In vitro	NA	Substrate of CYP3A4/5; no effect from CYP1A2, CYP2A6, CYP2B6, CYP2C19, CYP2C8, CYP2C9, CYP2D6 inhibitors	NA	No induction on CYP1A2, CYP2B6, CYP3A4/5; no inhibition on CYP1A2, CYP2B6, CYP2C8, CYP2C9, CYP2C19, CYP2D6; may be time-dependent inhibitor of CYP3A4	Concomitant use of strong CYP3A4 inhibitors should be avoided due to the potential for an increase in DM1 exposure and toxicity. If cannot be avoided, consider delaying TDM1 treatment until inhibitors are cleared. If cannot delay, closely monitor for adverse reactions
Inotuzumab ozogamicin [6, 24]	Calicheamicin	In vitro	P-gp substrate, but not for BCRP, OATP1B1, or OATP1B3	Non-enzymatic reduction; expect no effect from CYP or UGT perpetrators (inhibitors or inducers) on drug CL	Low potential to inhibit P-gp, BCRP, OATP1B1, OATP1B3, OAT1, OAT3, and OCT2	No inhibition on CYP450 and UGT at physiologic concentrations	No recommendation
Polatuzumab vedotin [10, 39]	MMAE	In vitro	P-gp substrate	Substrate of CYP3A	Not an inhibitor of P-gp	Time dependent inhibitor of CYP3A4; No inhibition on CYP1A2, CYP2B6, CYP2C8, CYP2C9, CYP2C19, or CYP2D6; No induction on major CYP enzymes	Concomitant use of strong CYP3A inhibitors or inducers has the potential to affect the exposure to MMAE
		PBPK model	NA	Fluconazole (CYP3A inhibitor) increased MMAE AUC by 45% and C_max_ by 18% Rifampin (CYP3A inducer) decreased AUC by 63% and C_max_ by 41%	NA	MMAE showed no impact on midazolam (CYP3A substrate) exposure	
Enfortumab vedotin [9, 20]	MMAE	In vitro	P-gp substrate, but not for BCRP, MRP2, OATP1B1/3, OCT2, and OAT1/3	Substrate of CYP3A	Not an inhibitor of BSEP, P-gp, BCRP, MRP2, OCT1, OCT2, OAT1, OAT3, OATP1B1, or OATP1B3	Mechanism-based inhibitor of CYP3A4/5; No of CYP1A2, CYP2B6, or CYP3A4/5	No dose adjustments are recommended for drug-drug interactions. Closely monitor for signs of toxicity in patients taking concomitant strong CYP3A4 inhibitors, as potentially increased systemic exposure of MMAE may increase the incidence or severity of PADCEV toxicity
Trastuzumab deruxtecan [7, 24]	DXd	In vitro	OATP1B1 substrate, but not for OATP1B3, MATE2-K, P-gp, MRP1 and BCRP	Substrate of CYP3A4	Low potential to inhibit OAT1, OAT3, OCT1, OCT2, OATP1B1, OATP1B3, MATE1, MATE2-K, P-gp, BCRP, or BSEP transporters	No inhibition of CYP1A2, CYP2B6, CYP2C8, CYP2C9, CYP2C19, CYP2D6 and CYP3A; No induction of CYP1A2, CYP2B6, or CYP3A	No dose adjustment is needed for patients who are co-administered with CYP3A, OATP1B or P-gp inhibitors
		Clinical study	Ritonavir (OATP1B/CYP3A inhibitor) increased steady-state AUC_0-17 days_ of fam-trastuzumab deruxtecan-nxki by 19% and DXd by 22%	Itraconazole (CYP3A inhibitor) increased steady-state AUC_0-17 days_ by 11% for ADC and 18% for DXd	NA	NA	

*MMAE* monomethyl auristatin E, *DM1* emtansine, *DXd* exactecan, *CL* clearance, *NA* not available, *PBPK* physiologically based pharmacokinetic model, *P-gp* P-glycoprotein, *BCRP* breast cancer resistance protein, *MRP2* multidrug resistance-associated protein 2, *OATP1B1* organic anion transporting polypeptide 1B1, *OATP1B3* organic anion transporting polypeptide, *OCT2* organic cation transporter 2, *OAT1* organic anion transporter 1, *OAT3* organic anion transporter 3, *CYP* cytochrome P450 enzymes, *BSEP* bile salt export pump, *UGT* uridine glucuronosyltransferase, *MATE1* multidrug and toxin extrusion protein 1, *MATE2-K* multidrug and toxin extrusion protein 2-K

Given low systemic concentrations of released payloads relative to its in vitro K_i_/IC_50_ values of metabolizing enzymes and/or transporters, the risk for a payload to be a perpetrator of metabolizing enzymes and/or transporters is considered to be low. As shown in Table 3, most of these assessments are based on the theoretical risk assessments using the in vitro DDI and clinical data, which often results in the labeling statement such as, “at clinical relevant concentrations, the payload has no or low potential to inhibit the CYP enzymes and/or transporters”. In vitro studies showed that MMAE and DM1 exhibited time-dependent and/or competitive inhibition of CYP3A with K_i_ values in the micromolar range, however, the systemic levels of MMAE and DM1 released after administration of brentuximab vedotin and T-DM1 at their clinically approved doses are only in the nanomolar range [22, 23]. Consistent with these observations, a dedicated clinical DDI study showed that co-administration of brentuximab vedotin did not affect exposure to midazolam, a sensitive CYP3A substrate [38]. PBPK modeling by integrating the in vitro DDI and clinical data further confirms the low risk of MMAE for being a perpetrator for CYP3A substrates. The prediction results were highlighted in polatuzumab vedotin prescribing information [10].

In contrast, the potential for a released payload to be a DDI victim still exists, which could possibly impact safety as these payloads are highly potent and typically have a narrow or even no therapeutic window. As shown in Table 3, three out of the four payloads for the approved ADCs are metabolized by CYP3A with the exception of calicheamicin. In the case of calicheamicin, it has been shown that *N*-acetyl gamma calicheamicin dimethyl hydrazide, the main circulating catabolite, is extensively metabolized, primarily via non-enzymatic reduction of the disulfide moiety, but not CYP enzymes, thus DDI risk for *N*-acetyl gamma calicheamicin dimethyl hydrazide as a victim of metabolizing enzymes is considered low and no additional assessment was conducted. Dedicated clinical studies were conducted for brentuximab vedotin and trastuzumab deruxtecan to assess the DDI risk for the released payload as a victim. Low magnitude of DDI interaction for MMAE and DXd was observed when co-administration with strong CYP3A inhibitors and inducers. Co-administration of trastuzumab deruxtecan with itraconazole (a strong CYP3A inhibitor) and ritonavir (a dual inhibitor of OATP1B/CYP3A) resulted in an 18% and 22%, respectively, increase in steady-state exposure of DXd [25]. The magnitude of these changes is not considered clinically meaningful. In the case of brentuximab vedotin, co-administration with ketoconazole, strong CYP3A inhibitor, and rifampin, strong CYP3A inducer, increased MMAE exposure by ~ 34% and decreased MMAE exposure by ~ 46%, respectively [38]. As increased exposure to MMAE may increase the risk of adverse reaction, close monitoring of adverse reactions is recommended when brentuximab vedotin is given concomitantly with strong CYP3A inhibitors [5]. Instead of conducting a clinical DDI study, polatuzumab vedotin, an MMAE-containing ADC with the same linker and payload as brentuximab vedotin, adopted a PBPK approach to project the magnitude of DDI with strong CYP3A inhibitors and inducers. The PBPK model was developed using in silico and in vitro data and in vivo ADME and pharmacokinetic data of MMAE and a vc-MMAE ADC and subsequently verified by the clinical DDI data of brentuximab vedotin [39]. The model projections were used to inform polatuzumab vedotin prescribing information. A slightly different approach was used for enfortumab vedotin, another MMAE-containing ADC, where its prescribing information simply refers to the clinical DDI results for brentuximab vedotin. For T-DM1, concomitant medication analysis with the Phase III pivotal clinical study showed that co-medication of CYP3A inhibitors and inducers does not result in any noticeable change in the pharmacokinetics of T-DM1 and DM1 [40]. However, given a dedicated clinical study was not conducted, caution language with detailed instructions was included in the T-DM1 label.

Assessing DDI risk associated with the mAb component of ADCs is often relatively rare since DDI involving mAbs are typically limited. Population PK approach is commonly used for such a DDI assessment. Population PK analysis of inotuzumab ozogamicin identified concomitant rituximab treatment as one of the significant covariates on inotuzumab ozogamicin clearance (CL decreased by 16%). Similarly, population PK analysis with polatuzumab vedotin, which is approved for the treatment of *r*/*r* DLBCL in combination with rituximab and bendamustine, showed that combination with rituximab had 24% higher acMMAE exposure (ie, AUC) and 37–40% lower exposure of unconjugated MMAE compared to patients receiving single-agent [26]. There was no apparent impact of bendamustine on polatuzumab vedotin and MMAE PK. It is worth noting that the magnitude of DDIs seen with concomitant medications was small and was not considered clinically relevant. Therefore, no dose adjustment is recommended for inotuzumab ozogamicin or polatuzumab vedotin in concomitant treatment with rituximab.

In summary, given the complex structure and unique PK characteristics of an ADC, risk-based DDI strategy by integrating both large and small-molecule components of an ADC is warranted to support the clinical development and approval of an ADC. Theoretical risk assessment using in vitro DDI and clinical data should be conducted based on FDA and EMA DDI guidelines. Depending on the level of risk, different approaches may be implemented to further assess the DDI potential. Modeling based approaches such as population PK and PBPK modeling, have become increasingly accepted and used to support DDI assessment and regulatory submission for ADCs.

## QTc assessment

According to the ICH E14 [41] guidelines, it is generally recommended to evaluate the potential of non-antiarrhythmic drugs, such as ADCs to prolong the QT/QTc intervals in clinical development. A thorough QT study for an ADC is usually not feasible due to safety concerns on cytotoxicity of released payloads in healthy subjects and ethical concerns regarding a placebo arm in cancer patients. As an alternative, a clinical study that incorporates many of the key components of the thorough QT study is usually needed for an ADC, especially when there is evidence suggesting that the small-molecule component of the ADC or its catabolites are present in human systemic circulation.

Table 4 summarizes the approaches and results of QT assessment for the seven approved ADCs. In general, these seven ADCs did not show clinically meaningful impact on QTc prolongation, which is somewhat expected as the mAb component of the ADC is unlikely to interact with the human Ether-à-go-go-Related Gene (hERG) channel and the low concentrations of circulating payload after ADC dosing is unlikely to inhibit hERG channels in vivo. A dedicated clinical QT study was conducted for four out of the seven ADCs. The study design for brentuximab vedotin, T-DM1, and trastuzumab deruxtecan are similar, which involved a dedicated QT study collecting triplicate 12-lead ECG data with time-matched PK samples in ~ 50 cancer patients at a single dose level (i.e., clinical approved dose for brentuximab vedotin and T-DM1; a dose higher than clinical approved dose for trastuzumab deruxtecan). Gemtuzumab ozogamicin dedicated QT study is still ongoing (*n* = 56, NCT03727750). In comparison, inotuzumab ozogamicin, polatuzumab vedotin and enfortumab vedotin adopted a slightly different approach, instead of conducting a dedicated QT study, high-quality triplicate 12-lead ECG and time-matched PK samples were integrated in existing clinical Phase I and/or Phase II studies. Data pooled from one or multiple studies with ~ 17–250 cancer patients were used for QT assessment. It was noted that the majority of the approved ADCs had QT evaluation during cycles 1 and 3 representative of first dose and steady-state kinetics, except for enfortumab vedotin. Due to enfortumab vedotin’s short half-life (3.4 days for ADC; 2.4 days for MMAE) and dosing on Days 1, 8 and 15 of a 28-day cycle (see Table 4), triplicate 12-lead ECGs were collected on days 1 and 3 and days 15 and 17 during the first 28-days cycle to capture the QTc effects at first dose and steady-state kinetics, respectively. Regardless of the study approaches, analysis of ECG data from clinical studies typically follows the ICH E14 [41] guidelines. For the seven approved ADCs, QT intervals corrected for heart rate using Frederica’s formula (QTcF) are commonly used in concentration-QTc analysis. Three analytes (i.e., ADC conjugate, total antibody and unconjugated payload) were included in the concentration-QTc analysis for T-DM1, inotuzumab ozogamicin, polatuzumab vedotin while two analytes (i.e., ADC conjugate and unconjugated payload) used for brentuximab vedotin, enfortumab vedotin, trastuzumab deruxtecan (Table 4).

**Table 4:** QT assessment and key findings for the seven approved ADCs

Drug	Analytes	Study Method	Dose(s) evaluated	Label recommendation
Gemtuzumab ozogamicin (ongoing) [4, 45]	NA	Phase 4 open label, single-arm clinical study in adult and pediatric patients with r/r CD33‑positive AML (*n* = 56)	3 mg/m^2^ IV given on days 1, 4, and 7 (3 doses) per cycle QTcF evaluated on days 1, 4 (cycle 1 only) and 7 of cycles 1 and 2	QT interval prolongation has been observed in patients treated with other drugs containing calicheamicin
Brentuximab vedotin [5, 46]	MMAE, ADC	Phase 1 open label, single-arm clinical study in CD30 + patients (*n* = 46)	1.8 mg/kg IV Q3W QTcF evaluated on days 2,3,4 of cycles 1 and 3	No clinically meaningful change in QTcF (< 10 ms)
Trastuzumab emtansine (T-DM1) [8, 50]	ADC, TAb, DM1	Phase 2 open label, single-arm clinical study in HER2 + mBC (*n* = 51)	3.6 mg/kg IV Q3W QTcF evaluated on days 1 and 8 (cycle 1 only) of cycles 1 and 3	No clinically meaningful change in QTcF (< 10 ms)
Inotuzumab ozogamicin [6, 51]	ADC, TAb, Cal	Data pooled from 3 clinical studies in CD22 + r/r B-cell ALL or NHL pts (*n* = 250)	1.2–1.8 mg/m^2^ in single and divided doses per 28-day cycle QTcF on predefined postdose before blood drawn for PK analysis (days 1, 2 [cycle 3 and 4 only], and 7 [cycle 3 and 4 only] of Cycles 1, 3, 4, and 6)	No clinically meaningful change in QTcF (< 10 ms)
Polatuzumab vedotin [10, 52]	MMAE, acMMAE, TAb	Data pooled from 2 open-label clinical studies in patients with B-cell malignancies (*n* = 209)	1–2.4 mg/kg IV Q3W QTcF evaluated on days 2, 3, 4 of cycles 1 and 3	No clinically meaningful change in QTcF (< 10 ms)
Enfortumab vedotin [9, 21]	ADC, MMAE	Data pooled from phase 1 clinical study in patients with locally advanced or metastatic UC (*n* = 17)	1.25 mg/kg IV given on days 1, 8, and 15 (3 doses) per cycle QTcF evaluated on days 1, 3, 15, 17	At the recommended dose, EV had no large QTc prolongation (> 20 ms)
Trastuzumab deruxtecan [7, 25]	ADC, DXd	Phase 1 open label, single-arm clinical study in patients with mBC (*n* = 51)	6.4 mg/kg Q3W QTcF during cycles 1 and 3	The administration of multiple doses of TD (6.4 mg/kg every 3 weeks, which is 1.2 times the recommended dosage) did not show large mean effect (i.e., > 20 ms) on the QTc interval in an open label, single-arm study in 51 patients with HER2-expressing mBC

*ADC* antibody–drug conjugate (measured as conjugated antibody), *acMMAE* conjugated MMAE (measured as conjugated payload), *MMAE* monomethyl auristatin E, *T-DM1* trastuzumab emtansine, *DM1* emtansine, *DXd* deruxtecan, *TAb* total antibody, *QTcF* QT interval corrected for heart rate using Fridericia’s formula, *PK* pharmacokinetics, *Q3W* every 3 weeks, *NA* not available, *UC* urothelial carcinoma, *IV* intravenous, *AML* acute myeloid leukemia, *mBC* metastatic breast cancer, *ALL* acute lymphocytic leukemia, *NHL* non-hodgkins lymphoma, *UC* urothelial cancer, *r/r* relapsed/refractory, *ms* milliseconds, *EV* enfortumab vedotin, *TD* trastuzumab deruxtecan

Overall, QTc risk for ADCs is expected to be low given the mAb component of the ADC and low levels of circulating payloads. Leveraging preclinical and clinical data such as in vitro hERG test, cardiac safety data in animals and the level of circulating payload, is important for developing appropriate ECG strategy in clinical studies. Additionally, ECG monitoring may not be warranted for ADCs with the circulating concentrations of the released payload similar or lower than those established as having no QT effect. Although dedicated QT studies have been conducted for the 4 approved ADCs, increasing trends showed that integrating high-quality ECG monitoring and exposure-QTc analysis to the existing phase I and/or II studies could be an effective way to assess overall risk and meet regulatory submission requirements.

## Exposure–response (ER) modeling

Given a relatively narrow therapeutic window of ADCs [13] compared to mAbs, exposure–response (ER) analysis plays a critical role for supporting Phase II/III dose selection, label dose justification and guidance of dose adjustment for ADCs. Gemtuzumab ozogamicin dose is one of the examples highlighting the importance of ER analysis for selecting appropriate dose and schedule. Gemtuzumab ozogamicin was first granted an accelerated approval in 2000 as a monotherapy with dose of 9 mg/m^2^ for the treatment of patients with CD33 positive acute myeloid leukemia, however, the sponsor withdrew gemtuzumab ozogamicin from the market in 2011 as the confirmative study failed to demonstrate better efficacy but showed higher rates of fatal hepatotoxicity and veno-occlusive disease (VOD). Exploratory ER analyses of gemtuzumab ozogamicin using data from single agent of 9 mg/m^2^ dose showed that the risk for VOD increases as Cmax after first dose of gemtuzumab ozogamicin increases, while exposure-efficacy (i.e., complete remission) relationship, however, was relatively flat for any exposure measure including *C*_max_ after first dose, indicating a fractionated lower dose may have the potential to reduce the risk for VOD but preserve the efficacy of gemtuzumab ozogamicin. Recent positive study read-out with fractionated dosing of 3 mg/m^2^ confirmed the above hypothesis and demonstrated improved clinical benefit with reduced VOD risk, thus leading to the re-approval of gemtuzumab ozogamicin in 2016 [42, 43].

One of unique features of ADC ER analysis which is different from other therapies, is that it requires comprehensive understanding which analyte(s) are the key drive for efficacy and safety due to the complex structure of ADCs. Based on the mechanism of action, ADC conjugate, measured as conjugated antibody or conjugated payload, is generally believed to be the key analyte of interest to drive safety and efficacy for an ADC. However, it is worth noting that released payloads are highly potent and may possibly pose a safety risk, exposures of unconjugated drug are sometimes included in the exposure-safety analysis. Table 5 summarizes the ER results for the seven approved ADC. Among the seven approved ADCs, four of the ADCs, namely brentuximab vedotin, polatuzumab vedotin, enfortumab vedotin and trastuzumab deruxtecan, included both ADC conjugate and unconjugated drug analytes in their ER analyses. A positive exposure-efficacy relationship with ADC conjugate exposure was consistently observed for the four ADCs, however, no apparent or negative exposure-efficacy relationship was observed for unconjugated drug exposure. In comparison, exposure-safety relationships for the four ADCs vary, depending on safety endpoints and analytes used in the analyses. For brentuximab vedotin and enfortumab vedotin, ADC conjugate exposure appeared to correlate better with safety than that of unconjugated drug. In the case of brentuximab vedotin, a positive exposure-safety relationship was observed with ADC conjugate exposure, but not with that of unconjugated drug, while for enfortumab vedotin, positive exposure-safety relationships were observed with exposure of both ADC conjugate and unconjugated drug, but the strengthen of exposure-safety relationship appears to be much weaker for unconjugated drug. For polatuzumab vedotin and trastuzumab deruxtecan, no consistent exposure-safety trends were observed; positive exposure-safety relationships were observed sparsely between some safety endpoints and exposure of ADC conjugate and/or unconjugated payload. For T-DM1, inotuzumab ozogamicin and gemtuzumab ozogamicin, only one analyte was used in their ER analyses. Specifically, ADC conjugate was used for ER analyses of T-DM1 and inotuzumab ozogamicin. For both ADCs, increased conjugate exposure appeared to be associated with improved efficacy (i.e., ORR, PFS, OS). No apparent positive exposure-safety relationship was observed with T-DM1 treatment (i.e., hepatotoxicity and thrombocytopenia), while a positive exposure-efficacy relationship was founded between inotuzumab ozogamicin and some of treatment-related AE (i.e., Grade 3 + thrombocytopenia and HEAB-assessed VOD). Given ADC conjugate was not measured for gemtuzumab ozogamicin, total antibody analyte was used for the ER analysis instead. Together, for most of the seven approved ADCs, the efficacy endpoints appear to correlate best with ADC conjugate compared to that of unconjugated payload. For safety outcomes, while ADC exposures were often correlated with AEs, unconjugated payload exposures may also be important for certain AEs. Total antibody analyte was usually not included in the ER analysis since there is a high correlation between conjugate and the total antibody exposures [44].

**Table 5:** Summary of exposure-efficacy and exposure-safety analyses for the seven approved ADCs

ADC	Analytes	Exposure-Efficacy					Exposure-Safety				
		Dose	Study Popu- lation	PK Parameter	Efficacy Endpoint	Key Findings^*^	Dose	Study Popu- lation	PK Parameter	Safety Endpoint	Key Findings*
Gemtuzumab ozogamicin [4, 28]	TAb	9 mg/m^2^ on days 1 and 15	AML	C_max,cycle1_, AUC_cycle1_,and AUC_avg_	CR	No apparent relationship for TAb exposure Consistent relationship across exposure metrics	9 mg/m^2^ on days 1 and 15	AML	C_max_ and AUC after first dose, and average AUC	Liver toxicity (AST, ALT, total bilirubin), VOD	Liver toxicity: No apparent relationship for TAb exposure VOD: positive and significant relationship for **TAb** exposure Consistent relationship across exposure metrics
Brentuximab vedotin (AA) [5, 22]	ADC, MMAE	1.8 mg/kg Q3W	r/r HL, ALCL	Average C_min,ss_	ORR	Positive trend with **ADC** and negative trend with MMAE	0.1–3.6 mg/kg Q3W	CD30 + hematological refractory malignancies	Average C_min,ss_	Grade 2 + PN event, Grade 3 + neutropenia, Grade 3 + thrombocytopenia	PN: positive trend with **ADC**, no relationship with MMAE Neutropenia: positive trend for **ADC**, negative trend for MMAE Thrombocytopenia: No apparent relationship for both analytes
Trastuzumab emtansine (T-DM1) [8, 23]	ADC only	3.6 mg/kg Q3W IV	HER2 + mBC	Model-predicted C_min,cycle1_	ORR, OS/PFS	ORR: positive and significant trend with **ADC** C_min, cycle1_ OS/PFS: positive and significant trend with **ADC** C_min, cycle 1_	3.6 mg/kg Q3W IV	HER2 + mBC	Model-predicted C_min,cycle1_	Grade 3 + hepatotoxicity, Grade 3 + AEs, Grade 3 + thrombocytopenia, dose adjustments, PN	Hepatotoxicity: negative trend with ADC C_min,cycle1_ Grade 3 + AEs: negative trend with ADC C_min, cycle1_ quartiles Thrombocytopenia and dose adjustments: no apparent relationship PN: not reported
							0.3–4.8 mg/kg Q3W IV	HER2 + refractory and mBC			
Inotuzumab ozogamicin [6, 24]	ADC only	1.2–1.8 mg/m^2^ on days 1,8 and 15 per 28-day cycle	CD22 + ALL	cAUC_cycle1_, cAUC_ss_, C_max,cycle1_ and C_avg_	CR/CRi, PFS, OS	CR/CRi: positive and significant trend with **ADC** C_avg_ and cAUCP1 PFS/OS: positive and significant trend with **ADC** C_avg_	1.2–1.8 mg/m2 on days 1,8 and 15 per 28-day cycle	CD22 + ALL	cAUC_cycle1_, log(AUC_cycle1_)_,_ cAUC_ss_, log(cAUC_ss_) C_max,cycle1_, log(C_max,cycle1_), log(C_avg_) and C_avg_	Grade 3 + thrombocytopenia, VOD, Grade 3 + neutropenia, liver toxicity (ALT, AST, total bilirubin), hepatic events	Thrombocytopenia: positive and significant relationship with **ADC** cAUC_cycle1_ VOD: positive and significant relationship with **ADC** cAUC_cycle1_, only in patients who did not undergo HSCT post inotuzumab ozogamicin treatment All other AEs: no apparent relationship
Polatuzumab vedotin (AA) [10, 26]	acMMAE (ADC), MMAE	Supporting Phase I/II studies: 0.1–2.4 mg/kg Pivotal study only: 1.8 mg/kg mg/kg Q3W	r/r DLCBL	AUC_cycle6_ (ADC only)	OS, ORR, PFS, time to relapse	Supporting Phase I/II studies: ORR: positive and significant trend with **ADC** AUC Pivotal study only OS: positive and significant trend with **ADC** All other efficacy endpoints: no significant relationship	0.1–2.4, including 1.8 mg/kg mg/kg Q3W	r/r DLCBL	AUC_cycle6_, C_max_ (ADC and MMAE)	Time to first dose modification due to AEs, dose intensity (pola, rtx, benda), Grade 2 + PN event, Grade 3 + anemia, Grade 3 + neutropenia, Grade 3 + infections and infestations, Grade 3 + thrombocytopenia, Grade 3 + diarrhea, Grade 3 + liver toxicity (AST, ALT, bilirubin)	Time to dose modification: Positive and significant trend with **acMMAE** C_max_ Dose intensity (pola only): Positive and significant trend with **acMMAE** AUC and C_max_ PN: Positive and significant trend with **acMMAE** AUC and C_max_ Anemia: Positive and significant trend with **MMAE** C_max_ and AUC All other AEs: no apparent relationship
Enfortumab vedotin (AA) [9, 21]	ADC, MMAE (monotherapy)	1.25 mg/kg on days 1, 8, and 15 per 28 day cycle	mUC	Model-predicted AUC_cycle1_, C_max,cycle1,_ C_min,cycle1_ (ADC and MMAE)	BOR, DOR, PFS, OS	BOR only: Positive trend with **ADC** exposure and negative trend with MMAE exposure Similar trend for all the 3 exposure metrics	0.5–1.25 mg/kg on days 1, 8, and 15 per 28 day cycle	Nectin-4-expressing malignant solid tumors	Model-predicted AUC_cycle1_, C_max,cycle1,_ C_min,cycle1_ (ADC and MMAE)	Grade 3 + TEAEs, Grade 3 + rash, Grade 3 + hyperglycemia, Grade 2 + PN event	G3 + TEAEs: positive trend with both **ADC and MMAE**, the trend, much weaker for MMAE Similar trend for all the 3 exposure metrics Other AEs: not reported
Trastuzumab deruxtecan (AA) 7, 25	ADC, DXd (monotherapy)	5.4, 6.4, 7.4 mg/kg Q3W	HER2 + mBC	AUC_cycle1_, C_max,cycle1_, C_min,cycle1_, AUC_ss_, C_max,ss_, C_min,ss_, and C_avg_ (ADC and DXd)	ORR, DOR, PFS	ORR: positive trend for all exposure metrics for **ADC**, only significant for C_avg_; no relationship with DXd DOR, PFS: no relationship for both ADC and DXd exposure	0.8–8.0 mg/kg Q3W	HER2 + mBC	AUC_cycle1_, C_max,cycle1_, C_min,cycle1_, AUC_ss_, C_max,ss_, C_min,ss_, and C_avg_ (ADC and DXd)	DISC AEs, dose reduction or interruption associated with AEs, INTER AEs, SAEs, any grade and Grade 3 + (AEs, anemia, neutropenia, thrombocytopenia, ILD), any grade and Grade 2 + and 3 + LVEF reductions	AE correlates with either **ADC** or **DXd** exposure Treatment emergent and grade 3 + LVEF reductions not analyzed

*ADC* antibody–drug conjugate (measured as conjugated antibody), *acMMAE* conjugated MMAE (measured as conjugated payload), *AEs* adverse events, *AESIs* adverse events of special interest, *ALBU* serum albumin concentration, *ALKP* serum alkaline phosphatase concentration, *ALL* acute lymphocytic leukemia, *cAUCP1* cumulative AUC in treatment cycle 1, *cAUC* cumulative AUC, *AST* aspartate aminotransferase concentration, *AUC*_*ss*_ area under the concentration–time curve at steady state, *AUC/time* time-averaged area under the concentration–time curve to the point of progression or censoring (efficacy) and over the duration of treatment or time of first occurrence of each AE (safety), *ALCL* anaplastic large-cell lymphoma, *ALT* alanine aminotransferase, *AML* acute myeloid leukemia, *BOR* best overall response, *BMAR* baseline percentage blast in bone marrow, *BLDH* baseline lactic dehydrogenase, *C*_*min*_ minimum concentration, *COV* covariate, *VOD* veno-occlusive disease, *C*_*max*_ maximum concentration in plasma, *CR* complete response, *CTCL* cutaneous t-cell lymphoma, *C*_*avg*_ average concentration defined as the cumulative AUC divided by its duration, *DISC AEs* discontinuation associated with adverse events, *DOR* duration of response, *DME* measurable disease, *T-DM1* trastuzumab emtansine, *DM1* emtansine, *DXd* deruxtecan, *ECOG* Eastern Cooperative Oncology Group performance status, *G3AEs* grade 3 and above (Grade ≥ 3) adverse events, *HER2 +:* human epidermal growth receptor 2 + , *HSCT* hematopoietic stem cell transplant dimension of target lesions, *ILD* interstitial lung disease, *INTER AEs* drug interruption associated with adverse events, *MMAE* monomethyl auristatin E, *mBC* metastatic breast cancer, *N* no, *NA* not available, *NDIS* number of disease site, *ORR* objective response rate, *PN* peripheral neuropathy, *PFS* progression free survival, *r/r HL* relapsed/refractory hodgkins lymphoma, *ORR4* objective response rate lasting at least 4 months, *r/r DLBCL* relapsed/refractory diffuse large B-cell lymphoma, *mUC* metastatic urothelial cancer, *SAEs* serious adverse events, *TAb* total antibody, *TMBD* baseline sum of longest, *Y* yes, *TEAEs* treatment-emergent adverse events, *AA* accelerated approval, *RTX* rituximab, *Pola* polatuzumab, *Benda* bendamustine, *LVEF* left ventricular ejection fraction

^*^The analyte associated with a positive trend key finding is bolded

It is worth noting that four out of the seven ADCs (i.e., gemtuzumab ozogamicin, brentuximab vedotin, T-DM1 and enfortumab vedotin) use the data from single dose level in the exposure-efficacy analysis given efficacy data is indication-specific and only one dose level is usually studied in the pivotal study. Similar to ER analysis of other cancer-targeting biologics, caution needs to be taken to interpret the ER results of an ADC when the analyses are performed with data with only single dose levels as the effect of disease severity on ADC exposure may confound ER relationship (i.e., a visual steep trend is seen when the true relationship is flat) [45, 46]. The exposure-safety, however, is less likely to be confounded as the safety data are often pooled across the multiple studies, dose levels and patient populations. As illustrated in Table 5, a range of clinically tested doses were included in the ER safety analysis for most of the seven approved ADCs, while only three out of the seven ADCs include multiple dose levels in the ER efficacy analyses.

In summary, ER analysis provided valuable information beyond dose confirmation of the clinically tested dosage regimen in the phase 3 studies. We have illustrated the impact of ER analyses of gemtuzumab ozogamicin to enable test a fractionated lower dose thus leading to the re-approval of gemtuzumab ozogamicin. Additionally, ER analyses could guide dose adjustment. For brentuximab vedotin, the positive ER relationships with peripheral neuropathy and neutropenia support the dose reduction recommendation from 1.8 to 1.2 mg/kg in the event of Grade 2 + peripheral neuropathy and Grade 4 + neutropenia [22]. Furthermore, ER analysis could be used to identify the appropriate therapeutic dose for phase 2. For trastuzumab deruxtecan, ER analysis identified two potential phase 2 doses of 5.4 and 6.4 mg/kg from phase 1 data and confirmed the final dose recommendation of 5.4 mg/kg in pivotal studies based on similar predicted ORR probability (ORR 90% CI 0.63 [0.55, 0.70] and 0.68 [0.58, 0.77] for 5.4 mg/kg and 6.4 mg/kg, respectively) and exposure-safety relationships with greater rate of AEs in the 6.4 mg/kg group compared to the 5.4 mg/kg group [25].

## Summary and future directions

ADCs represent a rapidly evolving area of oncology drug development and hold significant promise. The complex structure of ADCs poses unique challenges to clinical pharmacology strategy in supporting development and approval of ADCs, since it requires a quantitative understanding of the PK and PD properties of multiple different molecular species (e.g., ADC conjugate, total antibody and unconjugated payload) in the systemic circulation and/or tissues of interest (e.g., tumors). Integration of diverse clinical pharmacology approaches, ranging from dedicated clinical pharmacology studies (e.g., DDI, QTc, renal/hepatic impairment study) to mechanistic and/or empirical models (e.g., PBPK, population PK modeling for one- or two- analytes, exposure–response analysis) can provide insights into the PK, PD and ADME properties of an ADC and inform development decision and clinical dose and schedule selection (Fig. 1). An additional consideration for clinical development not discussed in this review includes the thorough assessment of immunogenicity on ADC PK, efficacy, and safety.

As the field continues to evolve, the selection of suitable ADC targets and the identification of a target population remain critical challenges. Efforts to further optimize “next-generation” ADCs using engineered antibodies, innovative linkers, conjugation methods, and novel payloads are rapidly advancing. Despite the great success of ADCs, it is worth noting that the therapeutic window for ADCs remains relatively narrow with the maximum tolerated dose (MTD) often reached before ADCs achieve the maximum efficacious dose. Additionally, the toxicities associated with the ADCs might dictate the number of treatment cycles that the patients can tolerate and often result in dose delay, dose reductions or study discontinuation [13]. The future success of ADCs in part will depend on our ability to overcome these developmental challenges, especially by developing clear strategies to optimize the dose and schedule of ADCs and identifying predictive biomarkers to assess response, optimize patient selection, and inform potential combination therapies.
